# Supplementary material for: Using machine learning of clinical data to diagnose COVID-19: a systematic review and meta-analysis
Source: BMC Med Inform Decis Mak. 2020 Sep 29;20:247. doi: 10.1186/s12911-020-01266-z (PMC7522928; doi:10.1186/s12911-020-01266-z)
Supplement: Supplementary file 2 — Additional file 2 : Table S2. Significativity of ANOVA tests for SOM. [file 12911_2020_1266_MOESM2_ESM.pdf]

Table S2: Significativity of ANOVA tests for SOM

Clinical Variable	Significativity
Age	***
Sex	***
Region	***
Community Transmission	**
Number of family members infected	**
Neutrophil x10 9 L	**
Neutrophil level	***
Serum levels of white blood cell x10 9 L	**
Serum levels of white blood cell level	***
Lymphocytes	***
Lymphocytes level	***
Platelets	**
C reactive protein levels	***
C reactive protein levels high low	***
Eosinophils	**
Red blood cells	
Hemoglobin	**
Procalcitonin ng dL	**
Duration of illness (Days)	***
Days to death	***
Days before symptoms appear (Incubation)	**
Number of CT scan published	**
CT scan results - Pos Neg	***
Number of chest x rays published	**
RT PCR results	***
X ray results Pos Neg	**
Risk factors (Diabetes, Hypertension, gene alleles) etc.	***
Smoking	***
Number of affected lobes	
GGO ground glass opacity	***
Before symptoms appear incubation	***
