# Supplementary material for: Using machine learning of clinical data to diagnose COVID-19: a systematic review and meta-analysis
Source: BMC Med Inform Decis Mak. 2020 Sep 29;20:247. doi: 10.1186/s12911-020-01266-z (PMC7522928; doi:10.1186/s12911-020-01266-z)
Supplement: Supplementary file 3 — Additional file 3 : Table S3. p-values of Kruskal-Wallis Tests across Superclusters. [file 12911_2020_1266_MOESM3_ESM.pdf]

Table S3: p-values of Kruskal-Wallis Tests across Superclusters

1	Age	4.18E-24
2	Sex	6.76E-11
3	Region	0.00464726
4	Neutrophil Levels	2.62E-26
5	Serum Levels of WBC	1.04E-29
6	Lymphocytes	2.61E-44
7	Lymphocyte Levels	1.12E-40
8	C Reactive Protein Levels	1.34E-31
9	C Reactive Protein High or Low	5.41E-32
10	Duration of Illness	6.64E-09
11	Days to Death	0.06458636
12	CT Scan Results	1.50E-17
13	RT PCR results	2.88E-10
14	Risk Factors	1.88E-10
15	Smoking	1.72E-06
16	Ground Glass Opacity	2.66E-15
17	Incubation before symptoms appear	0.81403554
18	Fever	3.58E-16
19	Coughing	3.80E-20
20	Shortness of Breath	4.18E-26
21	Sore Throat	7.88E-30
22	Nausea and Vomiting	0.01510208
23	Pregnant	5.64E-48
24	If pregnant did baby die	2.83E-44
25	If pregnant Premature Delivery	1.83E-21
26	Body Temperature	3.96E-06
27	Cancer	0.05768527
