# Supplementary figures and images for: Using machine learning of clinical data to diagnose COVID-19: a systematic review and meta-analysis
Source: BMC Med Inform Decis Mak. 2020 Sep 29;20:247. doi: 10.1186/s12911-020-01266-z (PMC7522928; doi:10.1186/s12911-020-01266-z)

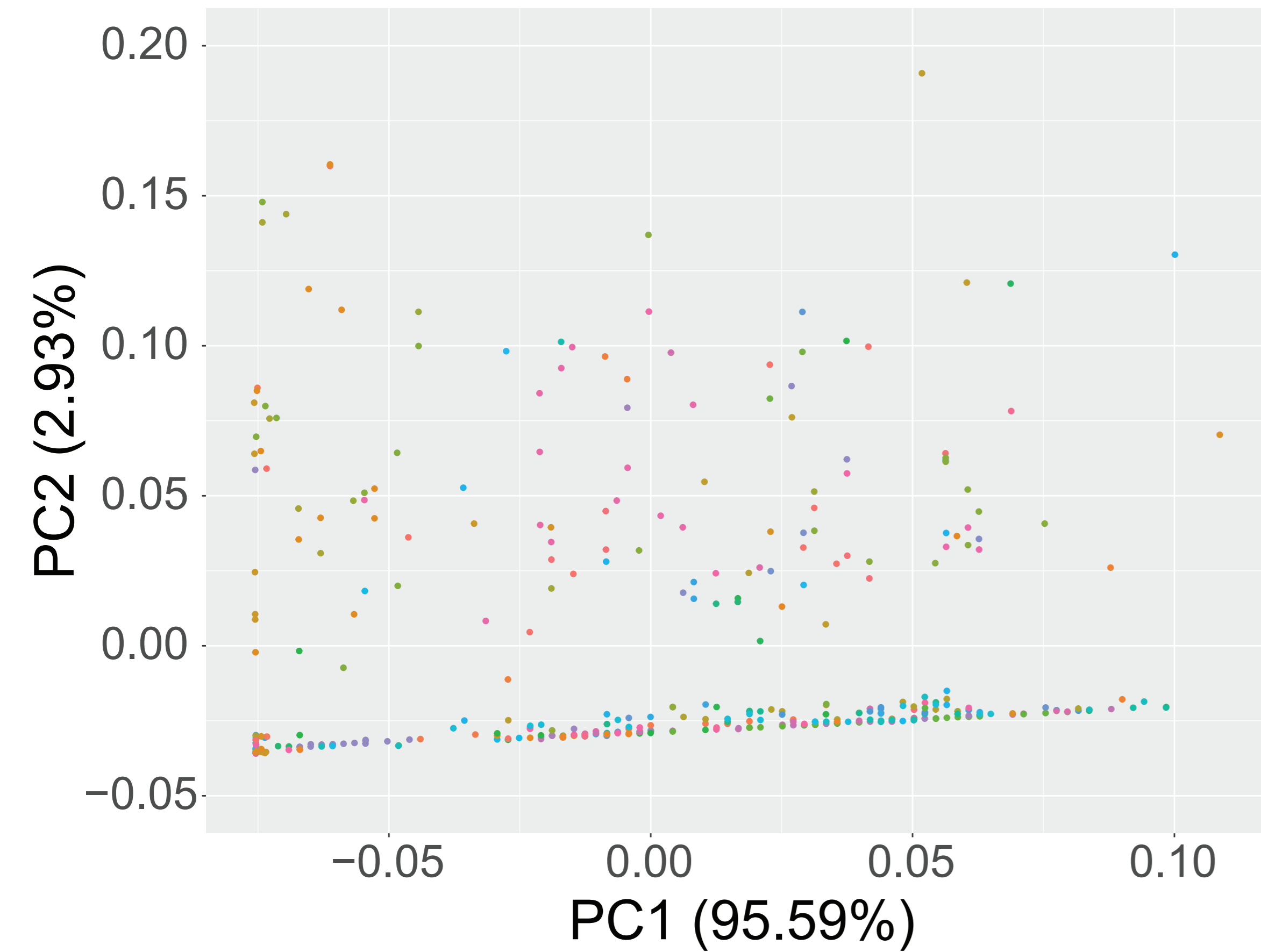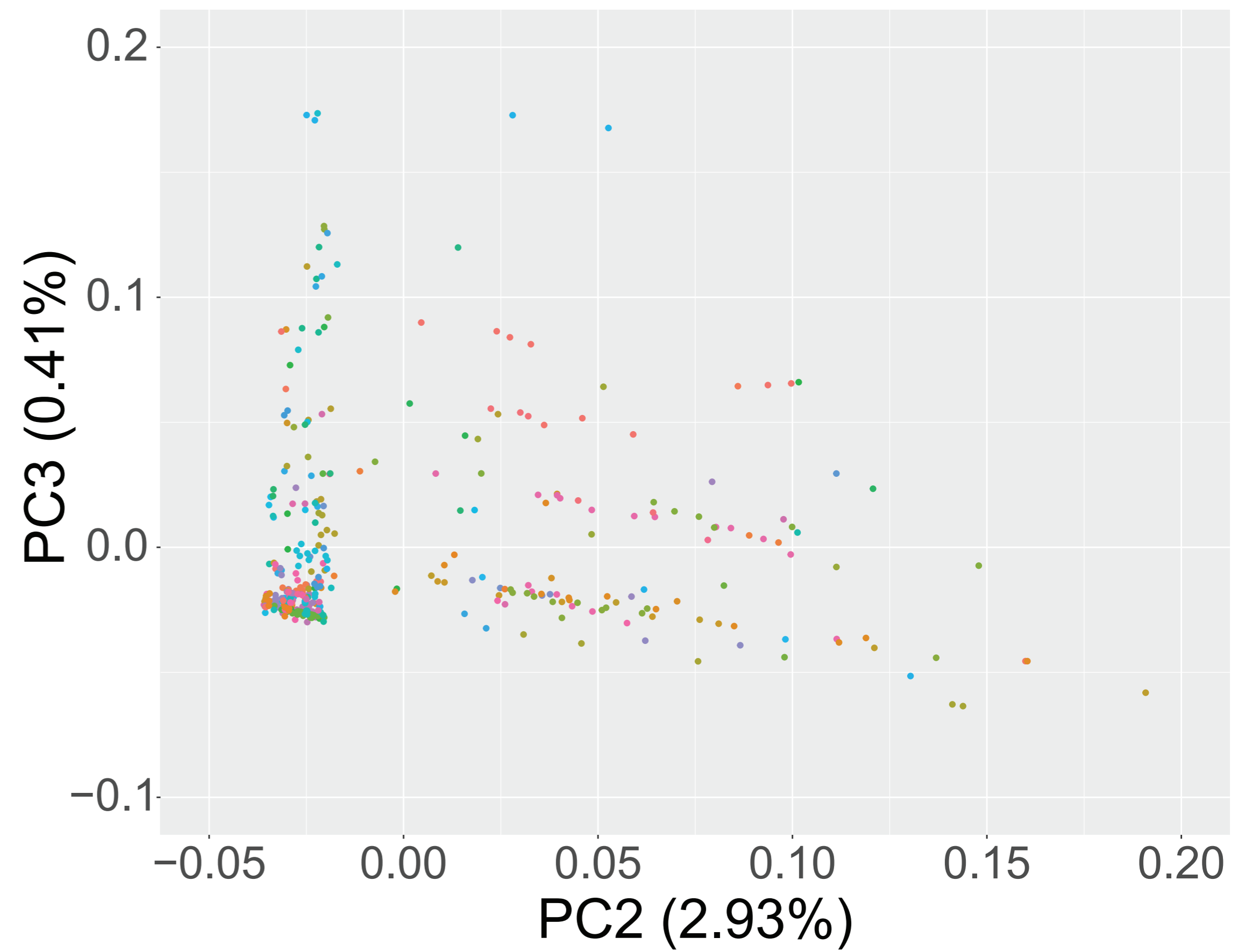

Supplement: Supplementary file 5 — Additional file 5: Figure S1. [file 12911_2020_1266_MOESM5_ESM.pdf]

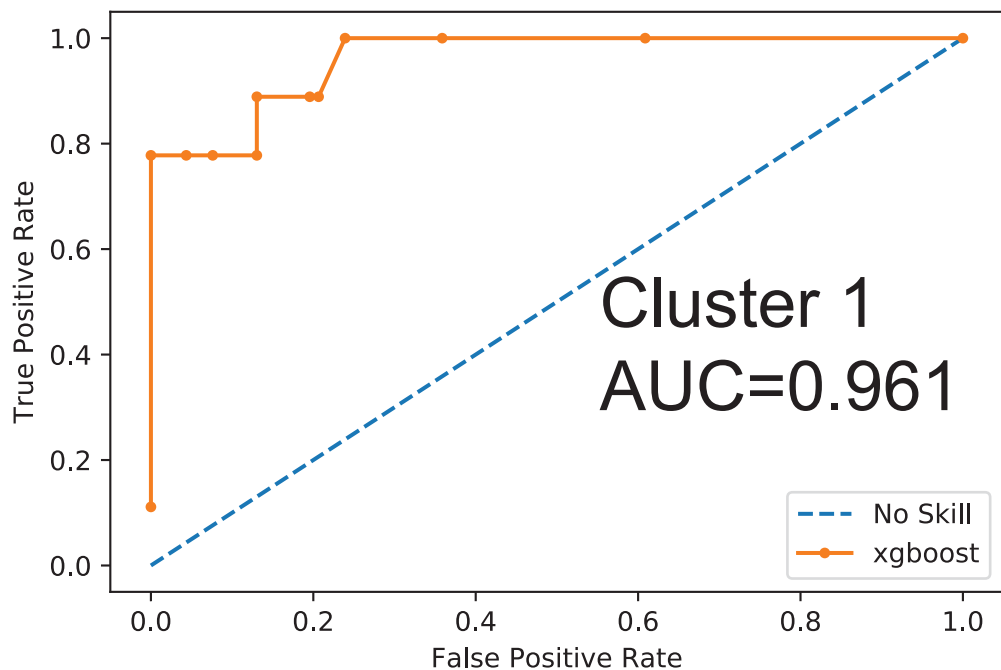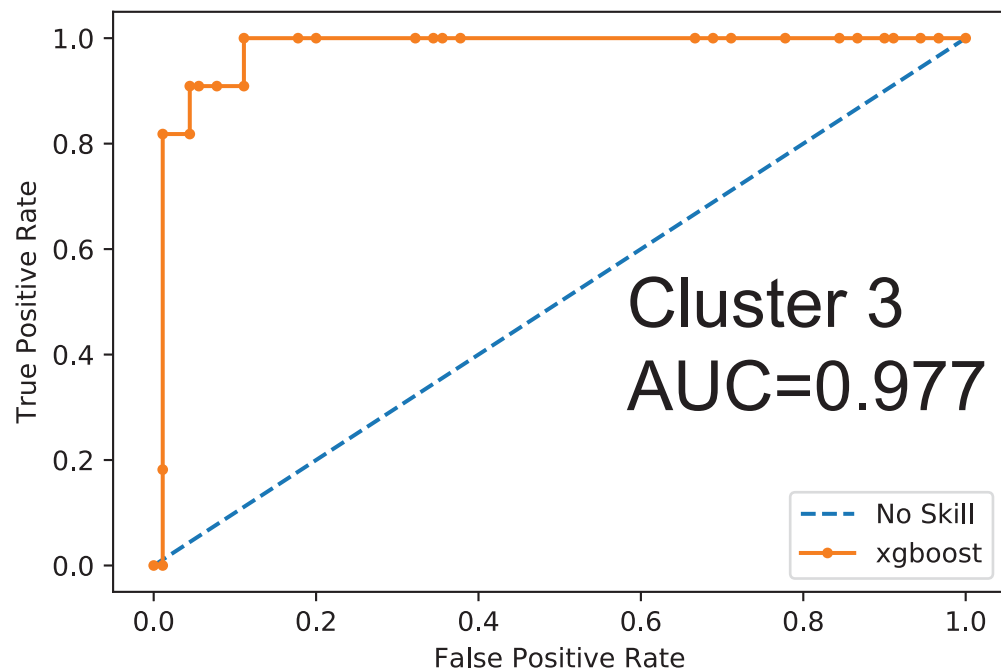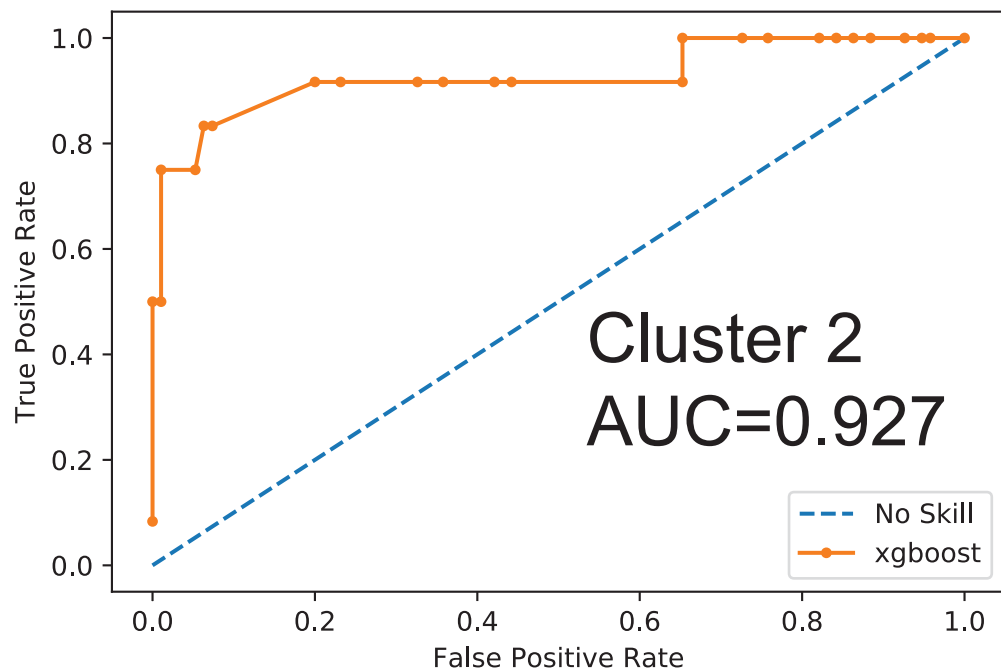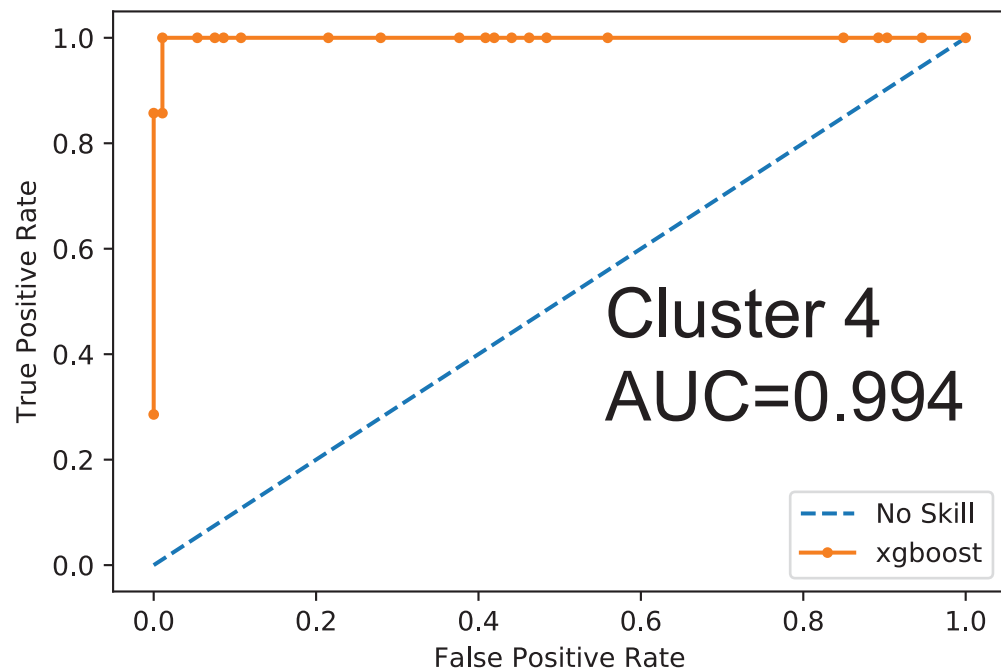

Supplement: Supplementary file 6 — Additional file 6: Figure S2. [file 12911_2020_1266_MOESM6_ESM.pdf]
